# Multidisciplinary team-guided combined endoscopic laparoscopic surgery for complex colonic lesions: a single-center retrospective cohort study

**DOI:** 10.1007/s00384-026-05131-9

**Published:** 2026-04-28

**Authors:** Mustafa Bulut, Svend Knuhtsen, Jens Ravn Eriksen, Lasse Bremholm, Ismail Gögenur

**Affiliations:** 1 Center for Surgical Science, Zealand University Hospital, Køge, Denmark; 2 Department of Surgery, Zealand University Hospital, Køge, Denmark; 3 Department of Clinical Medicine, University of Copenhagen, Copenhagen, Denmark

**Keywords:** Colon, Adenoma, Cancer, Polyp, Endoscopy, Laparoscopy, Multidisciplinary

## Abstract

**Purpose:**

Combined endoscopic laparoscopic surgery (CELS) is a minimally invasive alternative treatment for complex colonic polyps that can reduce surgical overtreatment. We report implementing a standardized treatment strategy with patients selected for CELS procedures via multidisciplinary team (MDT) conferences.

**Methods:**

This observational cohort study included 97 consecutive patients treated with CELS between 2016 and 2022. All cases were discussed by either a benign or malignant MDT. Two CELS techniques were employed: endoscopically assisted wedge resection (EA-WR) and laparoscopically assisted endoscopic mucosal resection (LA-EMR). Patients with suspected malignancies underwent step-up segmental resection (SR) if necessary. Primary outcomes were morbidity and mortality; secondary outcomes included adherence to MDT decisions, procedure durations, length of stay (LOS), histopathology, recurrence, and follow-up.

**Results:**

The approach decided by the MDT was unchanged in 81% of cases (79/97). Median age was 70 years and 43% were female. Lesions had a mean size of 31 mm and were predominantly located in the right colon. Technical success for lesion removal during the index procedure was 98% (95/97), with 93% completed by CELS alone. Median operative durations were shorter for EA-WR (52 min) and LA-EMR (73 min) than for SR (163 min, *p* < 0.001). Median LOS was 1 day for CELS and 5 days for SR (*p* < 0.001). Eleven patients (11.3%) experienced complications; four required re-interventions. Adenocarcinomas were found in 15 patients (15/97, 12.6%), with treatment individualized based on intraoperative and histological findings. The recurrence rate for benign lesions was 4%; these recurrences were exclusively in the LA-EMR group.

**Conclusion:**

An MDT-guided strategy incorporating CELS, with optional intraoperative step-up, is an individualized and organ-preserving approach to managing complex colonic lesions that minimizes unnecessary surgical resections. This strategy has the potential to improve clinical decision-making and should be validated in multicenter settings.

**Supplementary Information:**

The online version contains supplementary material available at 10.1007/s00384-026-05131-9.

## Background

Colorectal cancer (CRC) is the third most commonly diagnosed cancer worldwide and ranks second in cancer-related mortality [1]. The progression of colonic adenomas to carcinomas, along the adenoma-carcinoma sequence, necessitates the removal of these precancerous lesions as a standard clinical practice [2]. Consequently, many countries have initiated national colorectal screening programs to detect and prevent CRC [3]. Early removal of advanced adenomas may significantly reduce colon cancer rates and improve patient survival [4].

The introduction of colorectal screening programs has improved the detection of lesions. While many small polyps are treated with polypectomy during primary colonoscopy, larger or more advanced adenomas often require advanced endoscopic techniques, including endoscopic mucosal resection (EMR), endoscopic submucosal dissection (ESD), and endoscopic full-thickness resection (EFTR) [5]. Despite innovations in techniques, some complex lesions remain difficult to remove endoscopically due to their size, location, or submucosal invasion. Patients with such polyps may instead be offered radical surgical resection as a definitive treatment. Combined endoscopic laparoscopic surgery (CELS) has the potential to overcome these challenges and avoid overtreatment, whilst maintaining a minimally invasive surgical approach. Studies have shown that CELS can remove advanced adenomas safely and effectively, avoiding the need for segmental colonic resections, which are associated with risks such as anastomotic leakage [6].

For patients with CRC, discussion of treatment strategy by a multidisciplinary team (MDT) is mandatory. However, this approach is not standard for complex polyps, despite the presence of early cancers in 10–15% of specimens and a lack of consensus among experts regarding the best treatment modality [7, 8]. In the UK, structured MDT assessment of complex colorectal polyps is supported by a nationally defined framework, including a complex polyp minimum dataset to guide decision making and treatment strategy [9]. In contrast, Denmark currently lacks a national standardized MDT for assessing advanced adenomas. To address this gap, we adapted the existing CRC MDT structure to manage benign complex lesions.

This observational study evaluates the outcomes of a novel approach to treating complex colonic lesions via standardized MDT conferences and CELS.

## Methods

All patients scheduled for CELS between 2016 and 2022 at Zealand University Hospital, Denmark, were included, except those enrolled in a CELS trial for malignant lesions [10]. Consecutive inclusion was used to reduce selection bias. This study conforms to the Strengthening the Reporting of Observational Studies in Epidemiology (STROBE) guidelines for reporting observational studies.

The indications for CELS were benign colonic lesions deemed endoscopically unresectable by the referring endoscopist and small malignant tumors in patients who were high risk for surgery.

All cases were discussed either by a dedicated benign MDT or by the malignant colorectal MDT. The benign MDTs consisted of at least two colorectal surgeons and two expert endoscopists. Cases were assigned to the benign MDT when histopathology results demonstrated benign disease but the lesion was considered complex due to its size, morphology, location, suspected submucosal invasion, or to the likelihood of an incomplete or high-risk endoscopic resection procedure.

Cases were discussed by the malignant MDT when malignancy was confirmed or suspected. The additional specialists in the malignant MDT included a pathologist, an oncologist, and a radiologist.

### Multidisciplinary team conference

**Fig. 1:**
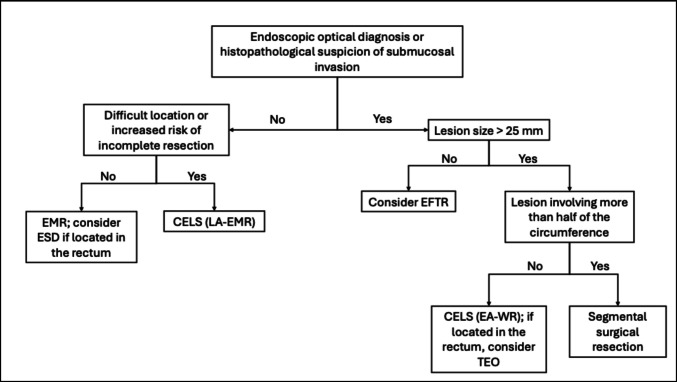
Multidisciplinary team-based algorithm used for treatment selection in patients with complex colorectal lesions. Decisions are based on optical diagnosis, histopathological assessment, lesion characteristics (including size, location, and circumferential involvement), and the estimated risk of incomplete resection or submucosal invasion. EMR: endoscopic mucosal resection, ESD: endoscopic submucosal dissection, CELS: combined endoscopic and laparoscopic surgery, LA-EMR: laparoscopic-assisted EMR, EA-WR: endoscopic-assisted wedge resection, EFTR: endoscopic full-thickness resection, TEO: transanal endoscopic operation

Images and video recordings from the index colonoscopy were reviewed during the MDT conference together with patient history, comorbidities, performance status, and available histopathological information. The endoscopists used the images and video for optical and morphological assessments of the lesions and to support MDT decision-making (Fig. 1).

**Fig. 2:**
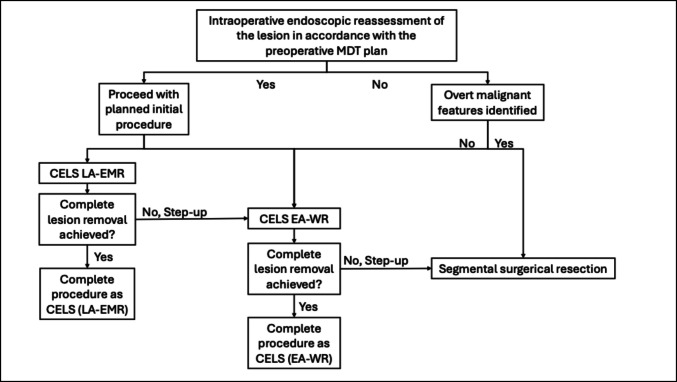
Intraoperative decision-making algorithm used during combined endoscopic and laparoscopic surgery (CELS). After reassessing the lesion intraoperatively in accordance with the preoperative MDT plan, the procedure proceeds as initially planned. If complete lesion removal is not achieved, step-up to endoscopic-assisted wedge resection (EA-WR) is undertaken. In cases where malignant features are identified or complete resection is not possible, conversion to segmental surgical resection is performed

In cases with confirmed or suspected malignancy, a thoracoabdominal CT scan was also presented at the MDT conference. If CELS was the treatment strategy selected, a procedure plan that included escalation to a more extensive intervention (“step-up”) if required was agreed (Fig. 2). Alternatively, the MDT could recommend standard EMR, ESD, EFTR, or conventional oncological surgery.

### Surgical approach

**Fig. 3:**
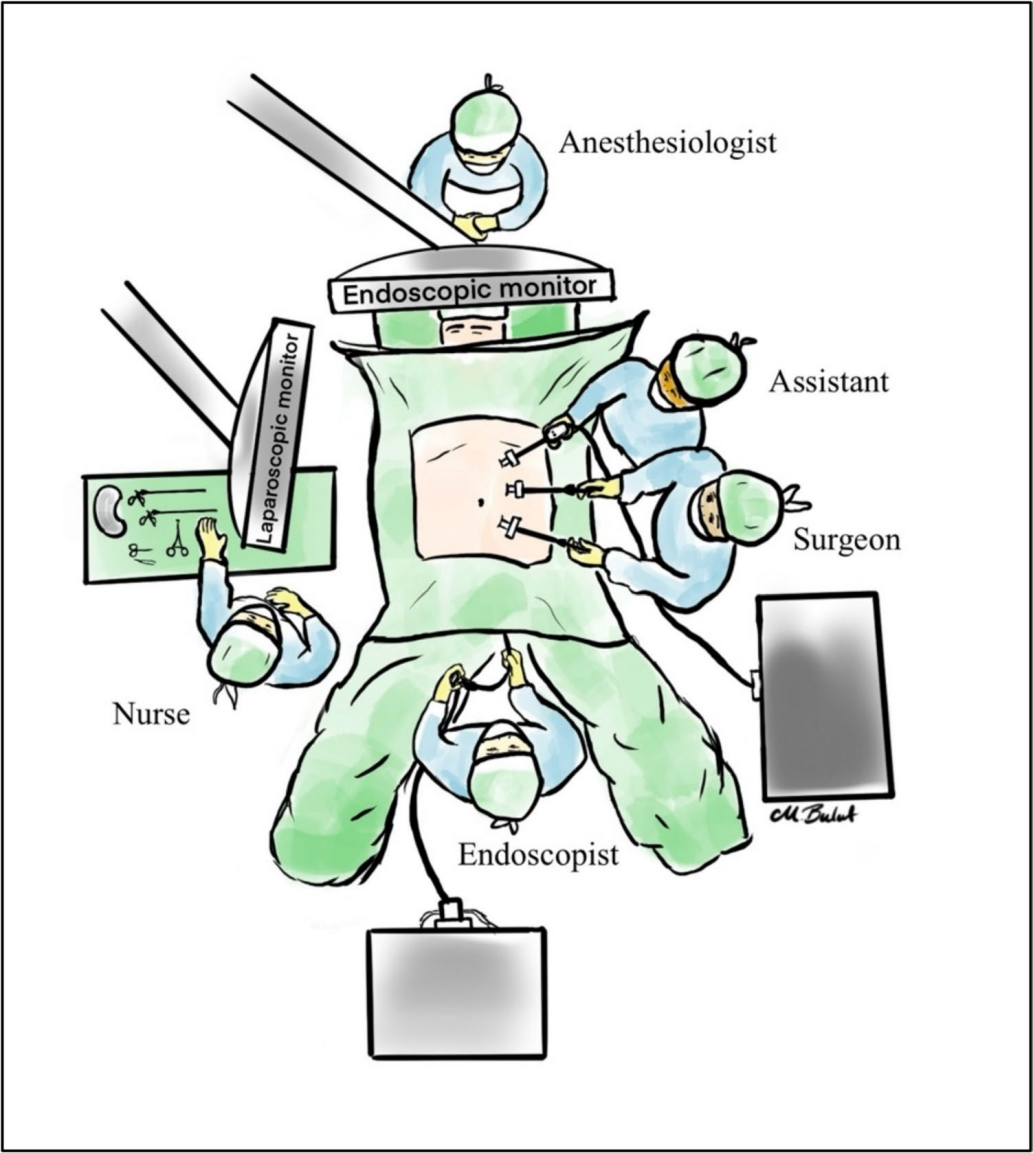
Team and room setup for the combined endoscopic and laparoscopic surgery, showing the positioning of personnel and monitors during removal of right-sided lesion in the colon

All patients underwent standard bowel preparation prior to the procedure. CELS procedures were performed under general anesthesia, with patients in a supine split-leg position. The operative setup is shown in Fig. 3, with endoscopic and laparoscopic views displayed on monitors that were visible to the entire team.

After creation of a pneumoperitoneum, three trocars were placed and the terminal ileum was clamped with an atraumatic grasper to prevent insufflation of the small intestine during colonoscope advancement.

Two types of CELS procedures were performed, either endoscopically assisted laparoscopic wedge resection (EA-WR) or laparoscopically assisted (LA)-EMR.

EA-WR involves locating the lesion endoscopically and precisely positioning a laparoscopic stapler to facilitate complete resection. In some cases, limited colon mobilization was necessary to optimize stapler positioning. During EA-WR, the resected specimen was opened intraoperatively along the stapled line to allow macroscopic assessment of resection margins before completion of the procedure.

LA-EMR is performed intraluminally. The laparoscopic team stretched or mobilized the colon to adjust the position of the endoscope. They also monitored endoscopic polyp resection from the serosa side of the bowel and managed any bowel perforations.

If there were concerns about the adequacy of resection during the procedure, a step-up approach was employed. For non-lifting areas during LA-EMR, the lesion was removed using EA-WR or surgical resection. Similarly, if malignancy was suspected during the procedure based on endoscopic optical diagnosis (including assessment of lesion morphology and pit/vessel patterns suggestive of invasive disease), a segmental resection (SR) was performed.

All endoscopic procedures were performed by one of three endoscopists, experienced in advanced therapeutic endoscopy. Surgical procedures were performed by colorectal surgeons, with at least one experienced board-certified colorectal oncological surgeon present during each case.

### Data analysis

All cases were consecutively registered in a CELS quality database. The data included were as follows: age; sex; ASA; comorbidities; anticoagulant medicine; preoperative size, type, and location of the lesion; MDT conclusion; surgical approach; operation duration; hospitalization; histopathology results; and complications within 30 days [11] and follow-up. Missing data were handled by complete-case analysis. Fisher’s exact tests and Kruskal–Wallis rank sum tests were used for comparative analysis and all statistical analyses were performed using R and RStudio software version 4.12.1 (R Core Team, 2024). Figures were created using the ggstatplot package [12–14].

### Ethics statement

The study was approved by the institutional ethical review board and registered with the Danish Data Protection Agency (REG-163–2020), in accordance with the relevant regulations. Informed consent was not required because the treatment was standard care for the department.

## Results

### Patient characteristics

In total, 97 consecutive patients were treated using the MDT conference and planned CELS strategy. There were 42 women and 55 men, with a median age of 70 years (range, 35–88), and a body mass index of 29 kg/m^2^ (range, 19–46). The majority of patients were classified as ASA physical status category II (65/97, 67%), and 68 of 97 (70%) had at least one registered comorbidity. Of these patients, 28 (29%) were taking anticoagulants, including warfarin, direct oral anticoagulants, and antiplatelets (Table 1).

**Table 1:** Patient demographics and clinical characteristics

	Overall (***N*** = 96)^a^	EA-WR (***N*** = 61)^a^	LA-EMR (***N*** = 28)^a^	SR (***N*** = 7)^a,*^	***p***-value^b^
Gender					0.572
Female	42 (44%)	29 (48%)	10 (36%)	3 (43%)	
Male	54 (56%)	32 (52%)	18 (64%)	4 (57%)	
Age	70 (35, 88)	69 (35, 86)	70 (52, 74)	75 (44, 88)	0.512
ASA					0.598
ASA I	13 (14%)	6 (9.8%)	6 (21%)	1 (14%)	
ASA II	64 (67%)	41 (67%)	18 (64%)	5 (71%)	
ASA III	19 (20%)	14 (23%)	4 (14%)	1 (14%)	
Anticoagulants					0.367
Antipatelets	16 (17%)	12 (20%)	4 (14%)	0 (0%)	
Direct oral anticoagulants	6 (6.3%)	6 (9.8%)	0 (0%)	0 (0%)	
Warfarin	6 (6.3%)	3 (4.9%)	3 (11%)	0 (0%)	
None	68 (71%)	40 (66%)	21 (75%)	7 (100%)	
Hypertension	45 (47%)	26 (43%)	14 (50%)	5 (71%)	0.369
Diabetes mellitus	17 (18%)	9 (15%)	6 (21%)	2 (29%)	0.434
Cardiovascular	20 (21%)	15 (25%)	5 (18%)	0 (0%)	0.398
COPD	17 (18%)	13 (21%)	2 (7.1%)	2 (29%)	0.130
BMI	29 (19,46)	28 (19, 46)	28 (20, 38)	31 (29, 39)	0.094

*EA-WR* endoscopically assisted laparoscopic wedge resection, *LA-EMR* laparoscopically assisted endoscopic mucosal resection, *SR* segmental resection

^*^Additionally, 3 patients underwent segmental resection as a salvage procedure during the observation period

^a^*n* (%); median (range)

^b^Fisher’s exact test; Kruskal–Wallis rank sum test

### Lesion characteristics

The lesions were distributed throughout the colon, but most (86/97, 89%) were localized in the right side of the colon (appendix, cecum, ascending colon, and hepatic flexure). Endoscopy showed that lesions had a mean size of 31 mm (4–100 mm) and histology was obtained in 72/97 (74%) cases before CELS (Table 2). Adenocarcinomas were confirmed in three cases, and CELS was selected as the preferred treatment option to prevent anemia and cancer progression in these patients. In all other instances, the lesions were either histologically benign or of unknown histology, due to intentional avoidance of biopsy or lack of endoscopic access in one case.

**Table 2:** Tumor, operative, and post-operative characteristics

	Overall (***N*** = 96)^a^	EA-WR (***N*** = 61)^a^	LA-EMR (***N*** = 28)^a^	SR (***N*** = 7)^a,*^	***p***-value^b^
Histology before					0.689
Adenocarcinoma	3 (3%)	3 (5%)	0 (0%)	0 (0%)	
Adenoma with high-grade dysplasia	15 (16%)	11 (18%)	3 (11%)	1 (14%)	
Adenoma with low-grade dysplasia	42 (44%)	23 (38%)	14 (50%)	5 (71%)	
Other	11 (11%)	9 (15%)	2 (7%)	0 (0%)	
Unknown	25 (26%)	15 (25%)	9 (32%)	1 (14%)	
Tumor size (mm)	31 (4, 100)	28 (4, 100)	37 (10, 80)	33 (15, 50)	0.246
Indication					0.898
Comorbidity	2 (2%)	2 (3%)	0 (0%)	0 (0%)	
Non-lifting	43 (45%)	26 (43%)	13 (46%)	4 (57%)	
Size or location	51 (53%)	33 (54%)	15 (54%)	3 (43%)	
Lesion type					0.942
Incomplete resection	25 (26%)	15 (25%)	8 (29%)	2 (29%)	
Late recurrence	7 (7%)	6 (10%)	1 (4%)	0 (0%)	
Other	1 (1%)	1 (2%)	0 (0%)	0 (0%)	
Untreated lesion	63 (66%)	39 (64%)	19 (68%)	5 (71%)	
Localization					
Appendix	23 (24%)	23 (38%)	0 (0%)	0 (0%)	
Ascending colon	20 (21%)	9 (15%)	8 (29%)	3 (43%)	
Cecum	37 (39%)	20 (33%)	14 (50%)	3 (43%)	
Descending colon	1 (1%)	1 (2%)	0 (0%)	0 (0%)	
Hepatic flexure	6 (6%)	3 (5%)	3 (11%)	0 (0%)	
Rectosigmoid colon	1 (1%)	0 (0%)	0 (0%)	1 (14%)	
Sigmoid colon	2 (2%)	1 (2%)	1 (4%)	0 (0%)	
Splenic flexure	3 (3%)	1 (2%)	2 (7%)	0 (0%)	
Transversum colon	3 (3%)	3 (5%)	0 (0%)	0 (0%)	
Operative time (minutes)	60 (14, 191)	52 (14, 177)	73 (38, 138)	163 (100, 191)	< 0.001
Postoperative complications	11 (11%)	7 (11%)	3 (11%)	1 (14%)	
Clavien-Dindo					
Grade I	4 (4%)	3 (5%)	1 (4%)	0 (0%)	
Grade II	2 (2%)	2 (3%)	0 (0%)	0 (0%)	
Grade IIIa	1 (1.0%)	0 (0%)	1 (4%)	0 (0%)	
Grade IIIb	3 (3%)	2 (3%)	0 (0%)	1 (14%)	
Grad V	1 (1%)	0 (0%)	1 (4%)	0 (0%)	
Length of stay (days)	1 (0, 16)	1 (0, 16)	1 (0, 3)	5 (1, 12)	< 0.001
Definitive histology					
Adenocarcinoma	15 (16%)	10 (16%)	0 (0%)	5 (71%)	
Adenoma with high-grade dysplasia	16 (17%)	6 (10%)	8 (29%)	2 (29%)	
Adenoma with low-grade dysplasia	49 (51%)	33 (54%)	16 (57%)	0 (0%)	
Other	6 (6%)	6 (10%)	0 (0%)	0 (0%)	
Sessile serrated adenoma with dysplasia	3 (3%)	2 (3%)	1 (4%)	0 (0%)	
Sessile serrated adenoma without dysplasia	7 (7%)	4 (6%)	3 (11%)	0 (0%)	
Lesion in diameter (mm)	25 (4, 80)	23 (4, 65)	28 (8, 80)	37 (14, 75)	0.141

*EA-WR* endoscopically assisted laparoscopic wedge resection, *LA-EMR* laparoscopically assisted endoscopic mucosal resection, *SR* segmental resection

^*^Additionally, 3 patients underwent segmental resection as a salvage procedure during the observation period

^a^*n* (%); median (range)

^b^Fisher’s exact test; Kruskal–Wallis rank sum test

### Multidisciplinary conference decisions

**Fig. 4:**
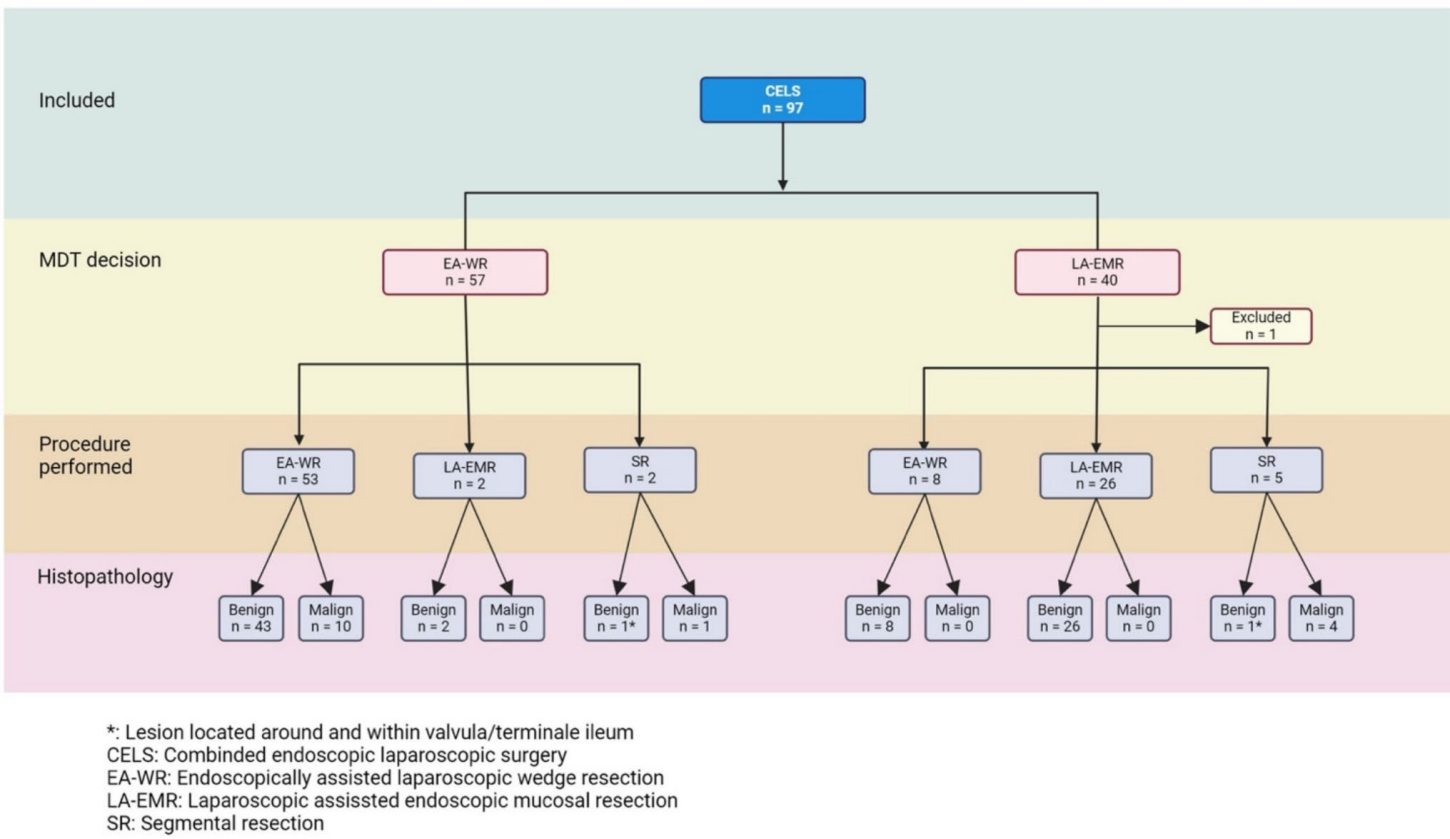
Flowchart showing MDT decision, procedures performed, and final histopathology outcomes for 97 patients undergoing CELS

All patients were evaluated by an MDT to select the initial approach. In total, 87 cases were evaluated by a benign MDT and the remaining 10 cases were evaluated by the malignant MDT. For 40 patients (41%), LA-EMR was selected as the initial approach, and this was unchanged for 26 of the 40 (65%) cases (Fig. 4). A further 8 cases (20%) continued with EA-WR and 5 (13%) continued with SR. Treatment of the remaining severely comorbid patient was aborted due to the inaccessible position of the lesion (the splenic flexure facing the retroperitoneum). Continuing the procedure would have involved comprehensive mobilization of the colon, which was inadvisable due to the surgical risk and benign histopathology (i.e., an adenoma with low-grade dysplasia).

In the group with EA-WR as the initial approach, there were 57/97 patients (59%), and in 53 cases (92%), the procedure was unchanged. Two procedures (4%) were changed to LA-EMR, whereas two were completed using SR.

Overall, the procedural plan allocated at the MDT conferences was unchanged in 79/97 procedures (81%). Deviations from the MDT plan were primarily due to intraoperative findings, such as non-lifting sign or suspected malignancy.

### Procedure outcomes

The technical success rate for complete lesion removal via CELS or a step-up SR during the same session was 95/97 (98%). The technical success rate for CELS alone was 90/97 (93%).

**Fig. 5:**
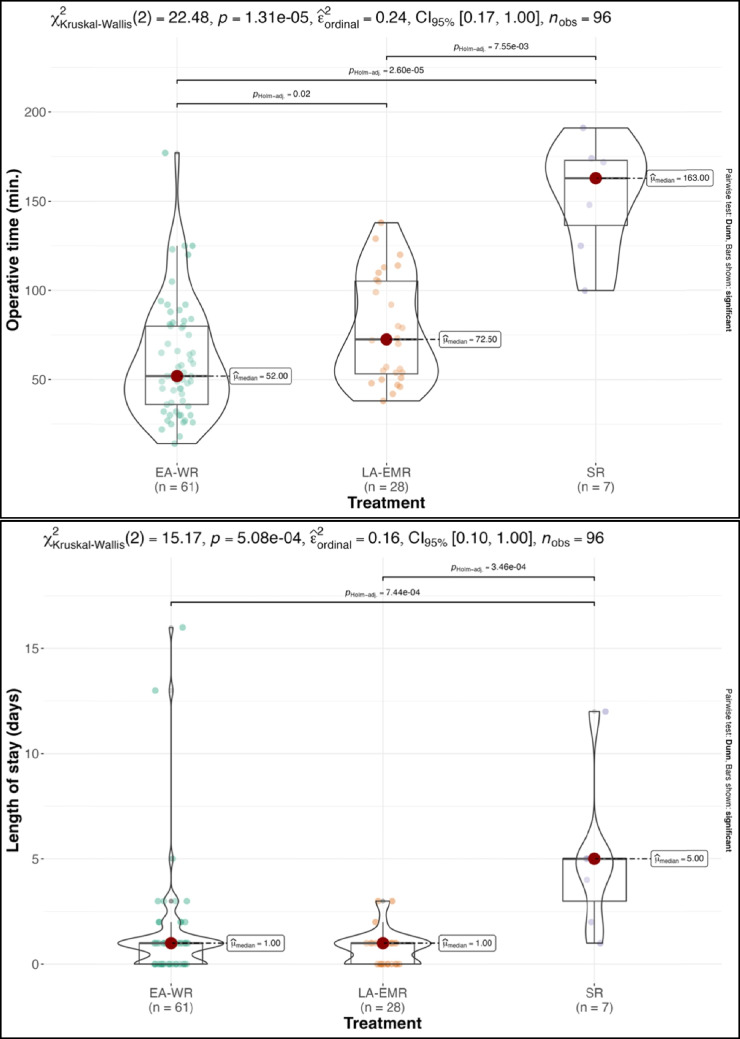
Comparison of operative time and length of stay across treatment groups. EA-WR: endoscopically assisted laparoscopic wedge resection, LA-EMR: laparoscopically assisted endoscopic mucosal resection, SR: segmental resection

The median operating durations for CELS procedures were 52 min (range, 14–177) and 73 min (range, 38–138) for EA-WR and LA-EMR, respectively (*p* = 0.02). Step-up operations that continued to SR had a longer median duration of 163 min (range, 100–191). No cases of conversion to laparotomy or need for colonic suture/repair occurred during LA-EMR. The operation durations for CELS were significantly shorter than those for SR (*p* < 0.001; Fig. 5).

### Postoperative outcomes

Patients that underwent CELS had a median length of stay (LOS) of 1 day (range, 0–16). The median LOS after SR was 5 days (range, 1–12). Patients undergoing CELS had a shorter LOS than those undergoing SR (*p* < 0.001), but there was no difference in LOS between the two CELS procedures (*p* = 0.23; Fig. 5).

A total of 11 patients (11.3%) experienced postoperative complications. Six cases involved Clavien–Dindo grade I–II complications. Four of these six cases involved complications due to subcutaneous hematomas at the port site, one was due to port infection, and one patient was treated with antibiotics. One grade IIIa complication occurred in a patient who underwent a diagnostic colonoscopy 3 days after LA-EMR due to hematochezia. The colonoscopy showed no further bleeding, but fibrin was present at the polypectomy site. Severe adverse events (Clavien–Dindo grades IIIb–V) were observed in 4 of the 97 patients (5%). All four of these patients required a second diagnostic laparoscopy, which revealed a port hernia, bleeding from the stapler line, and bowel perforation. Reoperation revealed an insufficient stapler line at the site of perforation in the patient with a perforated bowel. One patient developed severe aspiration during anesthesia, which resulted in cardiac arrest, when reoperation for port-site bleeding was performed.

### Definitive histopathology and follow-up

Follow-up data were available for all patients. The overall mean size of the CELS resected specimens was 25 mm. The mean size of the EA-WR specimens was 23 mm (range, 4–65 mm) and for the LA-EMR specimens this was 28 mm (range, 8–80 mm). The mean size of the segmental resected lesions was 37 mm (range, 14–75 mm).

Prior to the CELS procedures, 3 of the 97 patients (3%) had known malignant histopathology, but definitive histopathology revealed a further 12 cases of adenocarcinomas (Table 2).

**Fig. 6:**
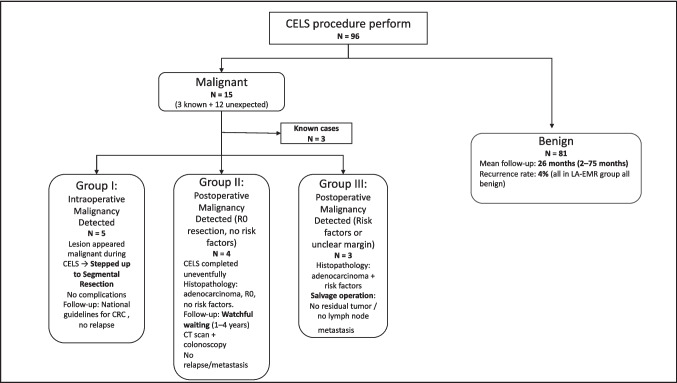
Follow-up and treatment pathway after CELS based on final histopathology and risk factors. Groups I–III illustrate different clinical pathways in patients with malignant histology

Malignant cases were categorized into three descriptive groups according to the timing of malignancy detection and subsequent management (Fig. 6).

Group I with five patients was defined by prior benign histopathology and assignment to CELS but these patients were optically assessed as having malignancies at the time of surgery. They were therefore stepped-up to SR without attempting CELS. The SR procedures were completed successfully and patients were followed-up in accordance with national guidelines.

Group II included four patients with prior benign histopathology who were treated with CELS (two with EA-WR and one with LA-EMR). The final histopathology results for these patients revealed malignancies but all four cases were resolved successfully with R0 resection margins and no additional histopathological risk factors. All chose to be followed up with watchful waiting. Follow-up times for these patients ranged from 1 to 4 years with CT scans and colonoscopies. There was no recurrence observed in any of these patients.

Group III included three patients who received CELS with EA-WR and had malignant histopathology revealing adenocarcinomas with R1 resection margins or histopathological risk factors, such as lymphovascular invasion. These patients underwent SR. Surgical specimens exhibited no residual tumors or lymph node metastases. The resected specimens exhibited 13–45 lymph nodes.

Overall, in patients with malignant histology (*n* = 12), the median follow-up duration was 36.5 months (range, 11–50 months; Table 3). For Group I (*n* = 5), median follow-up was 37 months (range, 11–50 months), with no local recurrence observed. For Group II (*n* = 4), median follow-up was 41.5 months (range, 15–48 months), with no reported recurrence. For Group III (*n* = 3), median follow-up was 36 months (range, 36–38 months), with no evidence of residual disease following salvage surgery.

**Table 3:** Clinical characteristics, management, and outcomes of the patients with malignant lesions

Group	ID	Preoperative histology	Initial approach/step-up	High-risk features	Subsequent management	Definitive histopathology after SR	Follow-up (months)
1	8	Unknown	EA-WR → SR	-	-	Adenocarcinoma	No recurrence (37)
1	23	Adenoma LGD	LA-EMR → SR	-	-	Adenocarcinoma	No recurrence (11)
1	29	Adenoma HGD	LA-EMR → SR	-	-	Adenocarcinoma	No recurrence (35)
1	34	Adenoma LGD	LA-EMR → SR	-	-	Adenocarcinoma	No recurrence (41)
1	78	Adenoma LGD	EA-WR → SR	-	-	Adenocarcinoma	No recurrence (50)
2	43	Adenoma HGD	EA-WR	R0; no high-risk features	Watchful waiting	-	No recurrence (47)
2	50	Unknown	LA-EMR	Rx	Watchful waiting	-	No recurrence (36)
2	77	Adenoma HGD	EA-WR	R0; sm2; no high-risk features	Watchful waiting	-	No recurrence (48)
2	95	Adenoma HGD	EA-WR	R0; no high-risk features; dMMR	Watchful waiting	-	No recurrence (15)
3	7	Adenoma HGD	EA-WR	R1 resection; venous invasion	Right hemicolectomy	No residual tumor; no lymph node metastases (45 nodes)	No recurrence (36)
3	46	Adenoma HGD	EA-WR	R1; lymphovascular invasion (T2)	Right hemicolectomy	No residual tumor; no lymph node metastases (13 nodes)	No recurrence (38)
3	61	Adenoma HGD	EA-WR	R0; sm2; nerve invasion	Right hemicolectomy	No residual tumor; no lymph node metastases (24 nodes)	No recurrence (36)

*EA-WR* endoscopically assisted laparoscopic wedge resection, *LA-EMR* laparoscopically assisted endoscopic mucosal resection, *SR* segmental resection, *LDG/HDG* low/high grade dysplasia, *Sm2* deep submucsal invasion, *dMMR* mismatch repair deficiency, *R0/R1* complete/incomplete microscopically resection, *Rx* resection margin not assessable

For benign cases, the mean follow-up period was 26 months (range, 2–75 months), with a 4% recurrence rate (4/97). Recurrences were observed exclusively in the LA-EMR group (4/28); no recurrences were observed after EA-WR (0/61). Comparison between EA-WR and LA-EMR using Fisher’s exact test demonstrated a statistically significant difference in recurrence rates (*p* = 0.008). Follow-up colonoscopies of benign cases after EA-WR found no residual adenomas. Follow-up colonoscopies of benign cases after LA-EMR revealed four cases with residual adenomas. Three of the cases had a 2–5 mm recurrent polyp. These were treated with snare polypectomy and subsequent follow-up colonoscopies showed no relapse. The final case involved a large (> 50 mm) benign polyp in the hepatic flexure, which occupied four-fifths of the lumen circumference. It was removed by LA-EMR. Follow-up colonoscopy 6 months later showed no residue, but recurrence was detected after 1.5 years. Due to the narrowing of the lumen from the previous EMR, no further CELS was attempted. Instead, laparoscopic SR was performed and the patient was discharged on postoperative day 2 after a successful recovery.

## Discussion

This study shows that a strategy involving MDT discussions and CELS offers a low-risk approach to managing complex colonic lesions, leads to high technical success rates (98%), reduces unnecessary SRs, shortens hospital stays, and decreases procedure durations. Importantly, the executed procedures were consistent with the MDT decisions in 81% of cases, demonstrating the reliability of the approach. In the remaining cases, intraoperative changes were primarily driven by lesion characteristics, such as non-lifting sign, unexpected malignant features, or challenging anatomical conditions, rather than misclassification at the MDT level. This highlights the importance of intraoperative reassessment and preplanned step-up contingencies. In this context, EA-WR, LA-EMR, and SR should be considered complementary techniques within a therapeutic spectrum rather than competing alternatives, allowing a tailored step-up approach based on intraoperative findings.

Although MDT conferences have been mandatory for CRC cases for several years, their application to cases with significant polyps that cannot be removed using standard endoscopic approaches is relatively new. The development of new techniques, such as EMR, ESD, EFTR, and CELS, means that platforms for discussing appropriate treatment plans need to include both surgeons and endoscopists, as well as radiologists and pathologists in some instances.

Multidisciplinary conferences have been implemented in other countries such as the UK, where they are nationally mandated. A UK study demonstrated an 82% reduction in the risk of unnecessary overtreatment and successful organ preservation by adopting systematic MDT evaluation [8]. Our study found that with MDTs, we were able to implement the planned procedure in 81% of cases. Moreover, we embedded the flexibility to step up, allowing us to adapt treatment successfully. Although the UK provides national guidelines for benign MDTs, to our knowledge there are no similar European or global guidelines. This emphasizes the importance of the data gathered in Denmark not only nationally but also as a model for other countries seeking to improve the management of benign colorectal lesions through structured MDT approaches [15].

This study included a total of 10 procedures (10%) that involved SR. Seven were performed during the primary procedure, and three (3%) were subsequent salvage procedures. The final histology of these lesions was generally adenocarcinoma, except for two cases of adenomas located around and within the valvula, which necessitated SR. These results are consistent with those from a multicenter Dutch study involving 118 colonic lesions that reported a need for subsequent salvage SR in 11% of cases [6]. In our setup, however, only 3% of cases required salvage SR, likely due to better patient selection facilitated by the MDT and the option for step-up SR.

The overall technical success rate for complete lesion removal, either as CELS or step-up SR, was 98%. However, the technical success rate for completing the procedure as CELS alone was 93%, which is consistent with reports from other studies [6].

In addition to being a minimally invasive procedure with organ-preserving benefits, CELS resulted in shorter operating times and LOS. In our study, the operating durations for both CELS techniques were significantly shorter than durations for laparoscopic segmental operations, which is consistent with findings from a study by Lascarides et al. [16]. Moreover, there was a statistically significant difference in operation duration between the LA-EMR and EA-WR techniques. Our data also revealed a significantly shorter hospital stay with CELS compared with laparoscopic SR (median of 1 versus 5 days), consistent with findings from other studies [17, 18].

A recent review found that postoperative complications after CELS ranged from 2.7 to 15.4% [19], which is similar to our reported rate of 11%, with severe complications in four cases requiring a second laparoscopic intervention. We did not observe any of the occlusions due to colonic stenosis after EA-WR that were described by Golda et al. [18]. To avoid this complication, we attempted to staple in a transverse direction and maintain the intraluminal positioning of the endoscope, with its tip proximal to the lesion to ensure the lumen is maintained. This is also important for lesions in the cecum, where the endoscope tip must be located in the terminal ileum to prevent occlusion of the bowel during stapling.

In our study, the mean follow-up period was 26 months (range, 2–75 months), with a 4% recurrence rate in benign cases, all within the LA-EMR group. There were no recurrences in the EA-WR group. Recurrence rates reported in the literature for LA-EMR range from 0 to 10% at a maximum of 65 months follow-up [20, 21]. For EA-WR, a 5% recurrence rate was reported in a recent prospective multicenter study [6]. Although recurrences were observed only in the LA-EMR group, there are not enough data to draw definitive conclusions. Large prospective studies will be needed to confirm these findings.

Surprisingly, only 12.6% of cases in our study exhibited adenocarcinomas, whereas a recent study in which lesions initially assessed as benign exhibited malignant histology reported a 20% incidence of adenocarcinomas [6]. Otherwise, our findings are consistent with those of previous studies [18, 22, 23].

Malignant cases were categorized into three clinically distinct groups based on the time when malignancy was detected and the histopathological risk profile. Group I included patients with benign or unknown preoperative histology with malignancies suspected intraoperatively, prompting immediate step-up to SR. Group II comprised patients with unexpected malignant histology identified after CELS was completed who had R0 resection margins, no high-risk histopathological features, and for whom watchful waiting was chosen. Group III included patients with unexpected malignant histology and high-risk features or uncertain resection margins on definitive histopathology, leading to subsequent salvage SR.

Treating adenocarcinomas in the colon with local resection is not standard practice due to concerns about unknown lymph node involvement. However, the risk of lymph node metastasis may be low in tumors without histopathological high-risk features [24]. Our treatment strategy for malignant cases involved a step-up approach for Group I patients, with oncological SR performed if malignancy was suspected during the CELS procedure. Group II consisted of patients with local R0 resection margins and no histopathological risk factors. These patients opted for watchful waiting and after 1–4 years of follow-up there was no endoscopic or CT evidence of relapses. Group III consisted of patients who underwent salvage operations and their histopathology results revealed no residual tumors or lymph node metastases in the final SR specimens. All three groups received appropriate treatment, although some patients required a second salvage operation.

Importantly, a significant proportion of benign cases would have undergone unnecessary SR if CELS had not been introduced. This claim is also discussed by Golda et al., who reported that 95.6% of cases in their segmental colectomy group underwent unnecessary colectomy and could have been treated by CELS instead [18].

Two different cost analyses that compared successful CELS with surgical management of benign colon polyps demonstrated that CELS resulted in lower overall costs. The most significant savings were attributed to shorter LOS and procedure durations [25, 26]. Although costs were not evaluated in our study, CELS did appear to be associated with shorter LOS and procedure durations, suggesting lower costs.

This study had some limitations. The retrospective design and the use of different treatment techniques limit generalizability. Additionally, this was a single-center study with a dedicated team of surgeons and endoscopists, which may introduce selection bias and reduce external validity in settings without similar expertise. Moreover, although follow-up was available for all patients, its duration varied, which may have resulted in underestimates for late recurrences, particularly after endoscopic treatment. However, all cases were consecutively included over a long period, and the follow-up, particularly for malignant cases, was comprehensive.

In conclusion, an MDT-guided strategy for managing complex colonic lesions facilitates low-risk individualized treatment selection, including organ-preserving strategies such as CELS. The integration of structured MDT decision-making with intraoperative step-up options provides a flexible and pragmatic framework that can reduce unnecessary SRs. This strategy may improve clinical pathways and decision-making for patients with complex benign and early malignant colonic lesions and can be integrated into routine clinical practice. Further multicenter studies are warranted to validate these findings and refine patient selection procedures.

## Supplementary Information

Below is the link to the electronic supplementary material.

ESM 1 (MP4.230 MB)

ESM 2 (DOCX.31.4 KB)

## Data Availability

The data that support the findings of this study are available from the corresponding author upon reasonable request.
